# Cancer as an Individual Risk Factor for Heart Failure: A Review of Literature

**DOI:** 10.7759/cureus.60592

**Published:** 2024-05-19

**Authors:** Marlon E Rivera Boadla, Nava R Sharma, Muhammad H Khan, Sakshi Khurana, Amit Gulati, Samuel Tan, Anupam Sharma, Amit Hooda, Prabal K. C.

**Affiliations:** 1 Internal Medicine, Maimonides Medical Center, New York, USA; 2 Medicine, Manipal College of Medical Science, Pokhara, NPL; 3 Radiology, New York Presbyterian-Columbia University Irving Medical Center, New York, USA; 4 Cardiology, Icahn School of Medicine at Mount Sinai, New York, USA; 5 Internal Medicine, Icahn School of Medicine at Mount Sinai, New York, USA; 6 Hematology and Oncology, Fortis Hospital, Noida, Noida, IND; 7 Interventional Cardiology, Icahn School of Medicine at Mount Sinai, New York, USA; 8 Internal Medicine, Rasuwa District Hospital, Kathmandu, NPL

**Keywords:** etiology of heart failure and treatment, risk factor for heart failure, cancer and heart failure, heart failure, cancer

## Abstract

The intricate relationship between cancer and cardiovascular diseases (CVD), notably heart failure (HF), is gaining attention in the medical field. This literature review explores the intricate interplay between cancer and CVD, particularly HF, emphasizing their significant impact on global mortality and comorbidity. While preventive measures have contributed to reducing their incidence, challenges persist in predicting and managing cancer-related complications. This review article delves into various risk factors associated with both cancer and HF, including lifestyle factors, genetic predispositions, and immune system dysregulation. It highlights emerging evidence suggesting a direct interaction between cancer and HF, with studies indicating an elevated risk of mortality from cancer in patients with HF and vice versa. Pathological mechanisms such as inflammation, oxidative stress, and tissue hypoxia are implicated in cancer-induced cardiac dysfunction, underscoring the need for comprehensive clinical investigations and ethical considerations in patient care. The review also discusses the potential role of biomarkers in risk assessment, early detection of cardiotoxicity, and understanding common pathophysiological links between cancer and HF, paving the way for multifaceted preventive and therapeutic approaches.

## Introduction and background

Heart failure (HF) is caused by various factors, some well-known and others still under study. In recent years, researchers have identified various risk factors and implemented strategies to lower the chances of developing HF. Nowadays, adequate control and supervision, as well as screening through indirect measures like follow-up of patients, has reduced or prevented the incidence and development of HF and cardiovascular disease (CVD) overall [1].

Nevertheless, cancer is known to represent a significant economic burden for the country. It is known to have a substantial impact not only on the person with the disease but also on their family, making it a socioeconomic problem [1,2]. Over the years, many strategies, like screening processes and vaccines, have been set to prevent different cancers. However, these efforts are often adequate for a minimal number of cancers. Overall, it is well known that cancer therapy can lead to many other problems, described as possible side effects and affecting distant organs than the one involved by the cancer itself [3].

Stoltzfus et al. described a study amongst 7.5 million cancer patients and identified that those who were diagnosed <40 years of age were more likely to die of heart disease when diagnosed with breast cancer or lymphoma; patients diagnosed >40 years of age were more likely to die from heart disease when diagnosed with prostate, lung, and colorectal cancers [4].

Cancer is known to be a hypermetabolic state and an activator of inflammation and immune cascade that theoretically can end up causing end-organ damage. On this basis, the possibility of developing different alterations in the human body is high. There has always been an association between HF and cancer treatment, such as breast cancer. However, there has also been an association between lymphoma, breast cancer, and HF even before treatment. Therefore, the question arises of whether there is a direct association of cancer as a sole risk factor for developing HF [2].

## Review

Risk factors for cancer

Over time, developing different mechanisms to study associations and the practical science of observation has led to an increased understanding of the various things that might lead to cancer. One of the very first associations was made in early 1913 when Marie Curie discovered the medical use of radiation and some of the adverse effects that could also carry their misuse [5].

Many have been established as risk factors associated with cancer; they can be carried out for generations, be part of the specific individual's genetic code, and are non-modifiable risk factors. On the other hand, scientific advances have identified multiple modifiable risk factors included in their majority conduct.

Modifiable

Risk factors that depend on the individual's conduct can be classified as modifiable. In general, many different lifestyles and adoption of behaviors lead to an increased risk of cancer, such as diet, deficient fiber intake, consumption of processed food, and high protein diet have been linked to increasing the risk of colon cancer. On the other hand, smoking has been linked to oral cancer and lung cancer. Certain acquired infections, such as *Helicobacter pylori*, have been linked to the incidence of gastric cancer [6,7].

The term modifiable means reversible, as the incidence of this cancer and its likelihood decreases as the inciting factor is stopped. Therefore, lifestyle changes are a significant factor in preventing cancer.

Non-modifiable

The genetic code has been studied since its discovery, and there's still a long road to fully understanding how to diagnose/treat diseases based on trying to modify it. Advances in technology have now allowed the treatment of many diseases by trying to reprogram the human genome by making available a deficient enzyme.

Nowadays, genetic testing has also been used to provide an alternative for cancer prevention, like the recognition of specific hereditary genes that are now well known to significantly increase the incidence of cancer, like the BRCA or MEN genes [8,9].

Risk factors for HF

Multiple causes have been identified and studied over the years to understand the pathophysiology of HF. Nowadays, guidelines are not just referred to treat but also to prevent. Nevertheless, it must be noted that just like any other disease, challenges are faced when it comes to factors that can't be removed from the equation when HF ensues.

Non-modifiable

Several different entities are included in this category. Even though US hospitalizations decreased until 2012, there has been a surge. The number of older adults has increased and is directly proportional to the incidence of HF; therefore, age plays an important role [2,9,10].

Primary and secondary hypertension can be debated on whether they are perceived as something that can't be changed. From the perspective that with old age, arterial stiffening takes a role and eventually decreases the compliance and mechanisms to adequately respond to changes in the body's needs, as well as hypertension in the setting of tumors such as pheochromocytoma [11].

On the other hand, diseases such as autoimmune, inherited genetic heart diseases, and infiltrative cardiac disease also fit this category as the mechanisms in which they develop are still under study, and so are the ways of preventing them [9,10].

Modifiable

Lifestyle modifications are the pillar of this category. Smoking has been proven to be the cause of CVD by itself, and its decrease is known to reduce the incidence of this phenomenon. Substance abuse has been an emerging cause as the development of drugs over the past 50 years has led to overall mortality predisposing to cardiac death [2,9]. The availability of different drugs and the increase in their consumption has contributed to the development of HF by itself [9,10]. Obesity, metabolic syndrome, and diabetes have been directly linked to contributing to the pathophysiology of HF, affecting homeostasis and wasting the body's regulation to excess energy that's preserved as fat and an increase in free radicals that eventually exert indirect and direct damage to the vessels and eventually the myocardium [7,12].

Pathophysiology of cancer

Formation of new blood vessels is a natural process critical for tumor growth and their later spread through different mechanisms. Most of the time, ischemia is one of the consequences of rapidly advancing cancers due to an inadequate response to the growth of the new tissue, leading to eventual necrosis. There are many factors involved in the development, such as the hypoxia-inducible factor (HIG), the fibroblast growth factor (FGF), vascular endothelial growth factor (VEGF), nitric oxide, and others [7]. Free radical formation from a continuous state of hypoperfusion and reperfusion to different organs in the body because of an increased metabolic rate due to cancer can also be seen. It can be hypothesized to be one of the causes of HF itself. We can also consider anaerobic processes mimicking or even producing minor myocardial infarctions due to hypoperfusion.

Immune activation

Nowadays, the immune system has been subject to new and innovative therapies based on its role in cancer development. Nevertheless, it is also known that these cells are also expressed in the myocytes. The increase in the incidence of HF with new drugs has been on the rise, and now, after observed adverse effects, prevention and screening have been implemented with the use of these drugs [7,13]. The involvement of different cells, such as macrophages, leucocytes, fibroblasts, antigen-presenting cells, T cells, and others, increases the production and expression of different growth factors (VEGF, PDGF) and interleukins (interleukin-1, interleukin-6, tumor necrosis factor-alpha), which drive tumor formation and development and eventual cardiac remodeling, precipitating the onset and progression of HF [1,13,14].

Discussion

Cancer and CVD have been recognized as some of the leading causes of death worldwide. In recent years, there has been increasing acknowledgment that both CVD and cancer are more prevalent among individuals who exhibit certain risk factors. Preventive measures have been instrumental in mitigating the incidence of these conditions [15]. However, while there has been a proliferation of research elucidating the risk factors associated with cancer, unlike HF, efforts to predict and reduce cancer risk have produced mixed results. Predicting individual cancer risk poses numerous challenges, including lengthy latency periods, a multitude of risk factors, each with a relatively minor impact, and an incomplete understanding of the causal pathways leading to cancer. Established risk factors include body mass index, race, age, genetics, sex, family history of cancer, history of tobacco use, and physical inactivity. However, the precise causal effects of many of these factors remain uncertain [16]. While identifying risk factors is crucial, understanding the impact of individual risk factors is essential for guiding physicians in stratifying individuals into low-, medium-, and high-risk categories and devising effective preventive strategies [16].

Patients frequently encounter challenges in adhering to medical advice, especially in urban settings where a significant portion of the population grapples with issues such as obesity, smoking, and sedentary lifestyles. Preventive measures primarily revolve around lifestyle adjustments. However, studies examining the effectiveness of supplementation, whether involving medications, vitamins, or dietary components, have shown limited success in reducing the incidence of new cancer cases [17]. A rare complication associated with cancer is pulmonary tumor thrombotic microangiopathy (PTTM), which has the potential to trigger pulmonary hypertension and subsequent right HF. This condition was described in 1990 by von Herbay et al. [18].

Gastric cancer, particularly the histologically mucinous, signet ring cells and poorly differentiated subtypes, is the primary malignancy commonly linked with PTTM. Although it is also associated with various carcinomas like lung, breast, ovarian, and bladder cancers, occurrences involving pancreatic cancer are relatively uncommon. To date, only four documented cases have been tied to pancreatic cancer. It's reasonable to infer that HF and cancer often manifest concurrently, given the considerable overlap in risk factors [19,20]. Indeed, there's substantial evidence pointing to a high prevalence of HF and cancer co-occurrence, with emerging indications suggesting a possible direct interaction between the two conditions [18].

A comprehensive analysis of a large pooled European cohort comprising 577,799 subjects over 12 years explored the potential correlation between hypertension and HF. The study revealed that with every 10 mmHg increase in blood pressure, there was an uptick in cancer incidence among men (hazard ratio (HR) 1.07, confidence interval (CI) 1.04-1.09). However, this association didn't attain statistical significance in women (HR 1.02, CI 1.00-1.05). Nevertheless, elevated blood pressure was linked to higher cancer-related mortality rates in both men and women (HR 1.12, CI 1.08-1.15, and HR 1.06, CI 1.02-1.11, respectively). These findings underscore a subtle yet noteworthy connection between hypertension and cancer [21].

In recent years, there has been growing recognition of the crucial roles played by innate immune and inflammatory signaling pathways in cardiac remodeling. Key molecules and receptors of the innate immune system are expressed in both cardiomyocytes and fibroblasts within the heart. Additionally, activation of the adaptive immune system, which depends on specific interactions between antigen-presenting cells and distinct antigen-specific receptors on T cells, significantly contributes to infarct healing and cardiac remodeling [22].

HF is characterized by a pronounced and sustained immunological and inflammatory response, which may impact the physiology of distant organs, including tumors. Immune system exaggerated reactions can lead to autoimmune-like diseases such as fulminant myocarditis, which carries a high mortality rate of nearly 50% [23]. As immune therapy increasingly benefits numerous cancer patients and its indications rapidly expand, these detrimental complications may unfortunately emerge as a new clinical challenge [24].

Various studies suggest that cancer instigates substantial tissue inflammation, oxidative stress, and localized neurohormonal activation, ultimately leading to cardiac wasting characterized by fibrosis and apoptosis [25]. According to Laplace's law, cardiac wasting, characterized by ventricular wall thinning, heightens ventricular wall stress, even without dilating. These changes impair cardiac function and may precipitate significant arrhythmias owing to electrical instability. Disturbances in the interstitial cardiac environment, resulting in myocardial cell death, could serve as a crucial substrate for the development of arrhythmias in cancer patients [26]. This hypothesis is supported by a study conducted by Shah et al., which investigated the correlation between cancer-induced cardiac changes and arrhythmias [27].

Ge et al. demonstrated that in a study involving 59,653, they evaluated the relationship between cancer and HF. In patients without cancer, the risk of mortality from cancer was higher (HR 1.36; 95% CI 1.09-1.69; P = 0.005); in patients with cancer, HF was associated with an elevated risk of death from cancer (HR 1.76; 95% CI 1.32-2.34; P < 0.001) [28].

‌Moreover, various crucial pathological mechanisms, such as cancer-induced pro-thrombotic conditions, disordered neovascularization, oxidative stress, and localized tissue hypoxia, could establish connections between cancer treatment and subsequent CVD [24,27]. Alterations within the microcirculation, which are also associated with numerous anti-cancer medications, may further contribute to the development of HF with preserved ejection fraction in individuals with cancer. Notably, evidence of cardiac wasting in advanced cancer has been documented in both preclinical models and human studies. This suggests a potential link between cancer progression and cardiac dysfunction [29].

Animal studies have demonstrated that onco-metabolites can induce cardiac dysfunction. Despite these discoveries, these concerns are frequently disregarded in clinical settings, particularly in advanced cancer stages, where patients typically receive palliative cancer-focused care, and systematic cardiac assessments are infrequent. Notably, CVD complications have been identified as a significant cause of mortality in cancer patients, often occurring after multi-organ failure and sepsis [30]. However, recent analyses suggest that CVD may be the second most common cause of death in individuals with cancer [31].

Patients with advanced cancer often exhibit symptoms and signs resembling HF, such as reduced mobility, congestion, dyspnea, and an elevated risk of sudden death [32]. In a study involving 177 autopsy reports of cancer patients, those with cachexia showed, on average, a 19% lower heart weight compared to those without cachexia. Additionally, in our investigation involving 120 patients diagnosed with non-small cell lung, colorectal, or pancreatic cancer, 8% experienced non-sustained ventricular tachycardia, with this condition being associated with a threefold increase in mortality [33].

Furthermore, a specific population analysis of cancer and HF is needed to elucidate the tight relationship between these two diseases completely. Identification of biomarkers particular to these two conditions is necessary for adequate surveillance and early diagnosis, posing a better understanding for further treatment and targeting specific pathways that might be involved [34,35].

Cardiac wasting is a prevalent occurrence in chronic diseases such as cancer and may arise from progressive loss of skeletal muscle and fat. This phenomenon can significantly impact cardiac function as part of the wasting disease cascade, highlighting the need for further clinical research. The identification of the cause of death in advanced cancer patients presents challenges, with ongoing uncertainty regarding event definitions in cancer-related fatalities. Therefore, it is crucial to undertake clinical investigations in this domain. When addressing cardiovascular issues in cancer patients, broader ethical considerations must be carefully deliberated.

## Conclusions

Cancer and CVD, particularly HF, stand out as among the most widely acknowledged contributors to mortality and comorbidity globally. While preventive measures have contributed to reducing the incidence of both conditions, the complexities of risk prediction and management persist, particularly in cancer-related complications. Despite advances in understanding the interplay between immune system dysregulation, tissue inflammation, and oxidative stress in cancer-induced cardiac dysfunction, clinical recognition and systematic investigation of these phenomena remain limited. Furthermore, the significance of cardiovascular complications in cancer-related mortality emphasizes the need for comprehensive clinical investigations and ethical considerations in patient care.

It is noteworthy that cancer, through its hypermetabolic state, can act as a sole risk factor for HF, further underscoring the intricate interplay between these two disease entities. Oncologists frequently refer patients for cardiological evaluation to assess risk and monitor treatment effects. Circulating biomarkers serve as a convenient and reproducible tool throughout the cardio-oncology journey, aiding in initial risk assessment, early detection of cardiotoxicity, and long-term follow-up. They may also reveal common pathophysiological links between cancer and HF, prompting multifaceted preventive and therapeutic approaches.
